# In-vitro and in-vivo study of the interference between Rift Valley fever virus (clone 13) and Sheeppox/Lumpy Skin disease viruses

**DOI:** 10.1038/s41598-021-91926-5

**Published:** 2021-06-11

**Authors:** N. Safini, Z. Bamouh, J. Hamdi, M. Jazouli, K. O. Tadlaoui, M. El Harrak

**Affiliations:** R&D Virology, MCI Santé Animale, Lot. 157, Z I, Sud-Ouest (ERAC), B.P. 278, 28810 Mohammedia, Morocco

**Keywords:** Biological techniques, Biotechnology, Cell biology, Immunology, Microbiology

## Abstract

Viral interference is a common occurrence that has been reported in cell culture in many cases. In the present study, viral interference between two capripox viruses (sheeppox SPPV and lumpy skin disease virus LSDV in cattle) with Rift Valley fever virus (RVFV) was investigated in vitro and in their natural hosts, sheep and cattle. A combination of SPPV/RVFV and LSDV/RVFV was used to co-infect susceptible cells and animals to detect potential competition. In-vitro interference was evaluated by estimating viral infectivity and copies of viral RNA by a qPCR during three serial passages in cell cultures, whereas in-vivo interference was assessed through antibody responses to vaccination. When lamb testis primary cells were infected with the mixture of capripox and RVFV, the replication of both SPPV and LSDV was inhibited by RVFV. In animals, SPPV/RVFV or LSDV/RVFV combinations inhibited the replication SPPV and LSDV and the antibody response following vaccination. The combined SPPV/RVFV did not protect sheep after challenging with the virulent strain of SPPV and the LSDV/RVFV did not induce interferon Gamma to LSDV, while immunological response to RVFV remain unaffected. Our goal was to assess this interference response to RVFV/capripoxviruses’ coinfection in order to develop effective combined live-attenuated vaccines as a control strategy for RVF and SPP/LSD diseases. Our findings indicated that this approach was not suitable for developing a combined SPPV/LSDV/RVFV vaccine candidate because of interference of replication and the immune response among these viruses.

## Introduction

Rift Valley fever virus (RVFV) is transmitted mainly by mosquitoes and causes severe Rift Valley fever (RVF) disease among humans and animals^1^. Outbreaks of RVF are often associated with favorable environmental drivers such as an elevated and widespread rainfall and flat topography that favors flooding^1^. As a member of the family *Phenuiviridae*, genus *Phlebovirus*, RVFV is an enveloped RNA virus that consist of three segments designated L, M and S of negative polarity^1,2^. Mature virions are released by budding in the Golgi compartment and replication occurs in the cytoplasm of infected cells^2^. Sheep and goat pox virus (SGPV) and lumpy skin disease virus (LSDV) of cattle belong to the genus *Capripoxvirus* of the family *Poxviridae*^3^. Poxviruses are large, enveloped, DNA viruses that replicate entirely in the cytoplasm^4^. Sheep and goat pox (SGP) and lumpy skin Disease (LSD) are acute contagious diseases that cause papule and pustule lesions of the skin and mucous membranes^5^. As a result, these diseases are responsible for enormous economic losses due to damage of the skin, reduced milk yield, mastitis, lowered fertility, and sometimes death due to secondary bacterial infections. After vaccination, animals have long-lasting immunity^6^.

Combined vaccines containing two or more viruses have been used in humans and veterinary medicine to reduce stress and vaccination cost. Measles, Rubella and mumps have been combined in one shot trivalent live vaccine for humans^7^. Sheep pox virus has been combined with small ruminant morbilli-virus (previously called peste des petits ruminants virus; PPRV) as one vaccine for small ruminants^8^ and a combined live vaccine for dogs affords immunity against canine distemper virus, hepatitis, and Parvovirus^9^. Combining multiple antigens has produced safe and efficacious vaccines, however, interference among antigens can be an obstacle for the development of such combined vaccines.

An effective way to establish solid herd immunity to selected diseases is through regular vaccination, but this is not widely practiced due to long inter-epidemic periods when there is no visible disease^10^. Combining the RVFV vaccine with other routinely used vaccines such as LSD of cattle or SGP of small ruminant may ensure regular vaccination and build herd immunity against RVFV infection for the new epidemics. In this study, we combined SPPV/RVFV and LSDV/RVFV to perform coinfection of susceptible cells and animals, sheep and cattle, to investigate the potential for interference of the replication of these viruses. In-vitro interference was evaluated based on estimates of infectivity determined the co-titration of SPPV/RVFV and LSDV/RVFV after three passages in lamb testis cell cultures and in-vivo interference was assessed based on the immunological response after co-injection of sheep and cattle with combination of these viruses.

## Materials and methods

### Viral strains

Attenuated RVFV clone 13 T, SPPV Romania strain and LSDV Neethling strain were used in this study^11–13^.

### Cell lines

Lamb testis (LT) primary cells were used for coinfection with SPPV/RVFV and LSDV/RVFV as they are susceptible to the three viruses. Preparation of primary LT cells was carried out according to previously described methods^11^. Vero cells (African green monkey kidney) were used for growth and titration of RVFV. Cells were cultured in Dulbecco's modified Eagle's medium (DMEM) with 10% fetal bovine serum (FBS) for growth and 1% FBS was used with the medium for preparing virus to inoculate the animals.

### Infection of cells

The susceptibility of LT and Vero cells to SPPV and RVFV was compared by infection using multiplicity of infection (MOI) 0.001, 0.01, 0.1 and 1, then titrated after harvest based on the development of generalized cytopathic effect (CPE). Coinfection was then performed by inoculating mixtures of SPPV/RVFV and LSDV/RVFV onto LT cells as following: for the first passage (P1), growth medium was removed and cells propagated in 25 cm^2^ flask were inoculated with LSDV or SPPV at fixed MOI then adsorbed for 45 min. Next, RVFV was inoculated onto cells at different MOI with media and without adsorption. The infected cells were examined daily. When cytopathic effect was 80%, the cells were harvested and frozen. The cell and virus suspensions were thawed at room temperature and the virus suspension was used for the next passage. In passage P2, infection of cells was performed with the P1 harvest without MOI calculation, the same was performed for P3. The infectivity titer and qPCR (Ct) were determined after each of the three passages.

### Virus titration

The infectivity titration of RVFV, SPPV and LSDV were achieved using an Immuno-fluorescence (IF) test. Ten-fold dilutions of each virus suspensions were prepared in the DMEM medium and inoculated onto LT cells and after 4 days incubation at 37 °C under 5% CO2, cells from each dilution were harvested and processed for IF assay. The LT cells co-infected with SPPV/RVFV or LSDV/RVFV were fixed with formalin (3.7%). Cells were then permeabilized with 0.1% Triton X-100 in phosphate-buffered saline (PBS) for 20 min. Monoclonal antibody anti-RVFV Gn-Gc (Alpha Diagnostics), LSDV and SPPV anti-sera internally produced were used as primary antibodies (polyclonal sheep serum anti-SPPV or bovine serum anti-LSDV). The binding of primary antibodies was detected by an anti-species IgG-fluorescein isothiocyanate (FITC) or Texas Red dye conjugate (from Bethyl). The highest dilution with fluorescence was considered as the virus titer, and calculated by Reed and Muench method^12^.

### Giemsa staining

Lamb testis cells were inoculated with RVFV and capripoxviruses and incubated for 4 days at 37° C under 5% CO2. The cell growth medium was discarded, and the cells were fixed with methanol for 15 min at room temperature. After removing methanol, cells were covered with Giemsa stain (4% v/v Giemsa-stain in double-distilled water) for 10 min, rinsed with several changes of tap water, air dried, and examined by light microscopy at 400 X.

### Quantitative real-time PCR

Nucleic acid RNA was extracted from 200 μl of RVFV and same amount of DNA of capripox viral-cell suspension samples using Isolate II genomic DNA kit (Bioline) and eluted in 100 μl of buffer according to the manufacturer’s instructions. Total viral RNA of RVFV was extracted from infected cells by using Isolate II RNA mini kit (Bioline). The extracted RNA was eluted in a volume of 60 µl of buffer and stored at − 20 °C until use. A quantitative real-time PCR (qPCR) TaqMan assay targeting ORF074 gene coding for the intracellular mature virion envelope protein P32 within SPPV and LSDV^13^, was used to determine viral genetic loads in clinical samples. Tests were performed in 96-well Optical Reaction Plates (Applied Biosystems), contained 10 μl Luna Universal probe QPCR Master Mix, 500 nM each capripoxvirus primer, 250 nM of capripoxvirus probe, 4 μl of template and nuclease free water to 20 μl. The reactions were run on the Quant Studio1 System (Applied Biosystems) using the following amplification program: 95 °C for 10 min; 45 cycles of 95 °C for 15 s and 60 °C for 1 min. The results were generated by the determination of the threshold cycle (Ct).

A one-step real time PCR assay was performed for detection of RVFV genome in a volume of 20 µl of RNA sample. The primer–probe set targeted the L-segment^14^. Tests were performed in 96-well Optical Reaction Plates (Applied Biosystems), that contained 10 μl SensiFAST Probe Lo-ROX one step Master Mix, 500 nM each primer, and 250 nM of fluorogenic probe, 4 μl of template. Reactions were run on the Quant Studio1 System (Applied Biosystems) using the following amplification program: reverse transcription at 45 °C for 15 min, initial denaturation at 95 °C for 2 min, 45 cycles with 95 °C for 15 s and 60 °C for 30 s. Fluorescence was read at the combined annealing-extension step at 60 °C.

### Animal experiments

Animal experiments were carried out in accordance with the international guidelines for the care and handling of experimental animals described in chapter 7.8 of the Terrestrial Animal Health Code and Directive 2010/63/UE of the European commission. The protocol was approved by the Internal Ethic Committee for animal experiment: Multi-chemical industry: MCI santé animale (Project number: 1M1604). Animals were separately housed into an insect proof BSL3 animal facility. Experimental design, minimisation of bias, sample size and statistical analyses of the in vivo experiments in the manuscript followed the recommendations in the ARRIVE guidelines.

Three groups of 10 sheep each, 4 to 6 months of age, were were vaccinated as follow: one group was inoculated with SPPV (4.0 (Tissue Culture Infectious Dose 50%) TCID_50_/dose), one group (n = 10) with RVFV (4.5 TCID_50_/dose) and one group (n = 10) with a mixture RVFV/SPPV (4.0 and 4.5 TCID_50_/dose, respectively).

Naive cattle from a local Moroccan breed, 6 to 8 months old were divided into five groups. Group 1 (n = 20) was inoculated with LSDV (10^3.5^ TCID_50_/dose), Group 2 with LSDV (n = 20) (10^4.5^ TCID_50_/dose), Group 3 with RVFV (n = 20) (10^4.5^ TCID_50_/dose), with Group 4 (n = 20) LSDV/RVFV (10^3.5^ and 10^4.5^ TCID_50_/dose respectively) and Group 5 (n = 20) co-infected with LSDV/RVFV (10^4.5^ and 10^4.5^ TCID_50_/dose respectively). Animals were observed daily for sign of illness and sera were collected weekly for serological analysis.

### Serological tests

Sera were screened for RVFV antibodies with a commercial competitive ELISA kit (RVF-ID Screen) from IDvet, France. Testing was performed according to the manufacturer’s instructions and results were read at a wavelength of 450 nm.

Virus neutralization tests were conducted as described in the OIE Manual (Chapters 2.7.11 and 2.7.14). This test is based on a serial ¼ dilutions of heat-inactivated sera and a constant amount of infectious virus (100 TCID_50_). The neutralizing antibody titer was calculated as the highest dilution to produce at least 90% neutralization in accordance to the Reed and Muench method^12^.

Interferon gamma (IFNG) test (Bovigam TB kit, France) was used to evaluate the IFNG levels. Animal blood samples were incubated overnight with positive control, a blank and LSDV. Samples were then tested for IFNG using a sandwich ELISA according to manufacturer’s instructions.

Challenge of vaccinated animals was performed with a virulent field Turkish Held SPPV strain (Hd2012) that had been passaged in LT cells and was provided during 2014 by the Institut veterinaire et agronomique in Rabat. Four of 10 tested sheep in group 1 that had been vaccinated with SPPV alone and four of 10 sheep in group 2 that had been vaccinated with SPPV/RVFV and two control naïve sheep (group 3) were challenged on day 35 post-vaccination (pv). The virus was administered by the intra-dermal (ID) route on the shaved flank of each animals as tenfold dilutions (10^−1^ to 10^−6^), five inoculation points per dilution. The average virus titer obtained for the vaccinated animals was compared with the average titer obtained for unvaccinated animals. Sheep were monitored daily for clinical signs, and the development of inflammation in each of the injection sites. The presence of any inflammation was considered positive for the virus titration. The average virus titer of G1 and G2 were compared with the titer obtained in the unvaccinated animals and the difference between the two, expressed in log_10_, represented the protection index^15^. The control animals were euthanized when severe generalized signs of illness were observed.

### Statistical analysis

All results were calculated and presented as the means ± standard error of the mean obtained from triplicate tests. The statistical significance was determined by one-way or two-way analysis of variance . P values of < 0.05 were considered as statistical significance.

## Results

### Interference SPPV/RVFV and LSDV/RVFV study on cell culture

**Figure 1:**
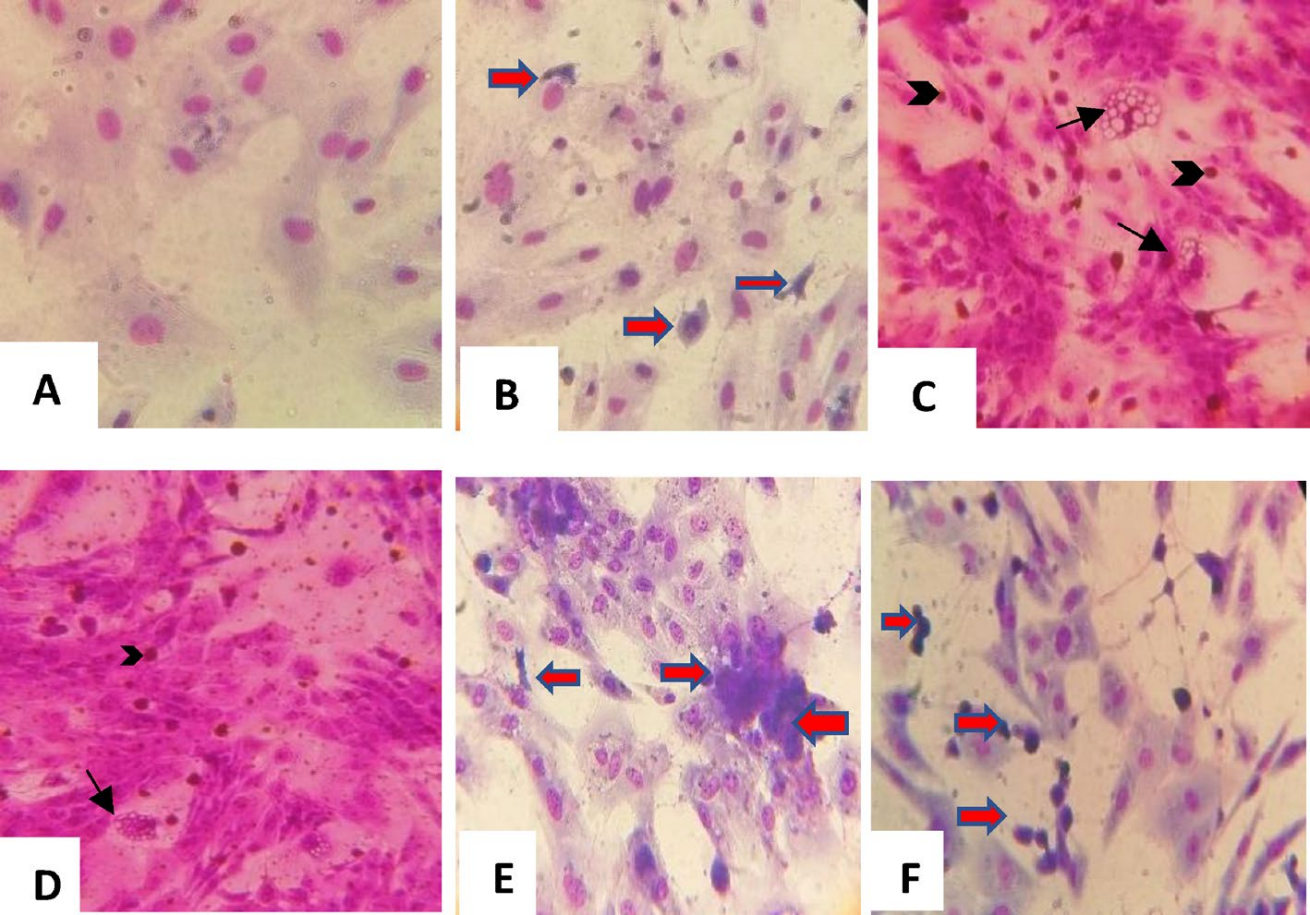
Giemsa staining of RVFV, SPPV and LSDV viruses for coinfection of LT cell culture (× 400). (**A**) Mock cells. (**B**) RVFV cytopathic effect: RVFV necrotic foci (red arrows). (**C**) SPPV effect (**D**) LSDV, (**E**) SPPV/RVFV 0.01/0.01 coinfection of LT cells and (**F**) LSDV/RVFV coinfection. Capripoxvirus induced cellular damage. Arrows showing capripox virus-induced vacuoles in cells and intracytoplasmic eosinophilic inclusion bodies (Arrowheads).

The susceptibility of LT cells to SPPV, LSDV and RVFV infection was evaluated by the appearance of CPE and the level of virus accumulation. When inoculated separately, RVFV induced necrotic cells that were quickly detached and released new virions in the supernatant that is why when we fixed the cells, as most of the remaining cells were either uninfected or those still present as necrotic foci (Fig. 1B) after a few hours’ post-inoculation (pi) of LT cells at a MOI 0.01 compared to mock inoculated cells (Fig. 1). The SPPV/LSDV induced vacuoles in cells and intracytoplasmic eosinophilic inclusion bodies (Fig. 1C and D) (typical capripox CPE profile). These bodies were slowly spread on the cell monolayer, whereas RVFV CPE was characterized by dead floating cells that infected the LT cell monolayer much faster than pox viruses. When the SPPV/LSDV were combined with RVFV clone 13 T at similar MOI, necrotic foci consisted of the major observed CPE. Indeed, in co-infected LT cells with SPPV/LSDV and RVFV, we observed also foci of necrotic cells (Fig. 1E and F). When the SPPV/LSDV MOI was higher than that of RVFV, typical pox CPE was observed on days 1 or 2 pi while RVFV CPE was observed 3 to 4 days pi.

**Figure 2:**
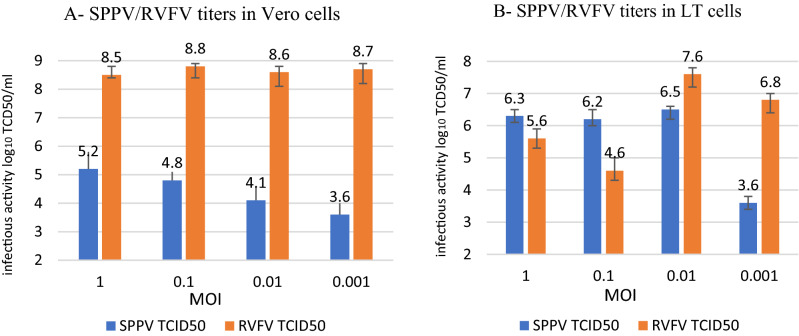
Infectivity titers of SPPV and RVFV co-cultured in Vero cells (**A**) and in LT cells (**B**) at different MOI 1, 0.1, 0.01 and 0.001. Data shown are means of at least 3 independent experiments.

The titer of SPPV/LSDV on Vero cells was low for all tested MOI (10^3.6^ to 10^5.2^ TCID_50_/ml), while the titer of RVFV reached 10^8.8^ TCID_50_/ml (data not shown for LSDV). This is suggesting that Vero cells were not susceptible to SPPV replication (Fig. 2A). In fact, when coinfecting with the 2 viruses in Vero cells, RVFV replication "overcame" SPPV's. In LT cells, the infectivity titer of the SPPV using high MOIs (1 and 0.1) was between 10^6.2^ and 10^6.5^ TCID_50_/ml, and surprisingly, the infectivity titer for RVFV was low for higher MOI: 1 and 0.1 (10^4.6^ to 10^5.6^ TCID_50_/ml) (Fig. 2B). Similarly, at low MOI (0.001), SPPV titer did not exceed 10^3.6^ TCID_50_/ml in both cell lines (Fig. 2). However, at low MOI = 0.001, RVFV infectivity titer reached10^6.8^ TCID_50_/ml in LT cells (Fig. 2B). Indeed, when LT cells were inoculated with similar higher MOI (1 and 0.1) for both viruses, SPPV replication dominated LT cells rather than RVFV (10^6.3^ and 10^5.6^ TCID_50_/ml for SPPV and RVFV respectively). The highest titers were obtained in LT cells at MOI 0.01 for both viruses SPPV and RVFV (10^6.5^ and 10^7.6^ TCID_50_/ml respectively) (Fig. 2B). Since LT cells were also susceptible to RVFV infection, subsequent coinfection experiments were conducted in these cells. These differences were statistically significant (p = 0.0025) based on results of the three different experiments.

**Figure 3:**
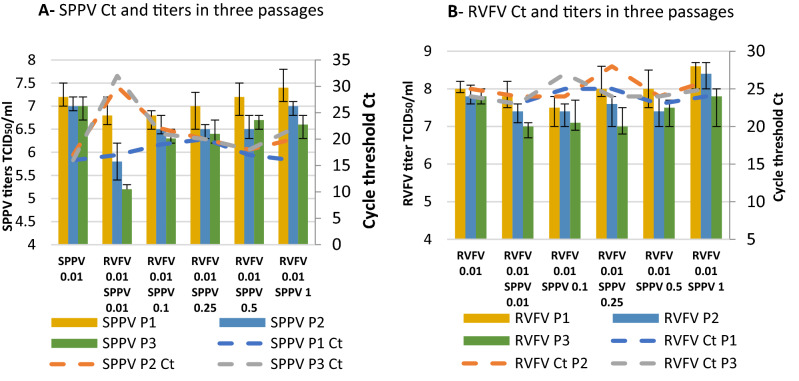
Infectivity titration (TCID_50_/ml) and viral genome copies by qPCR (threshold cycles: Ct) of SPPV (**A**) and RVFV (**B**) after coinfection in three successive passages (P1, P2 and P3). Infection was performed in (LT cells with different MOIs (RVFV/SPPV: 0.01/0.01; 0.01/0.1; 0.01/0.25; 0.01/0.5; 0.01/1). Data shown are means of at least three independent experiments.

**Figure 4:**
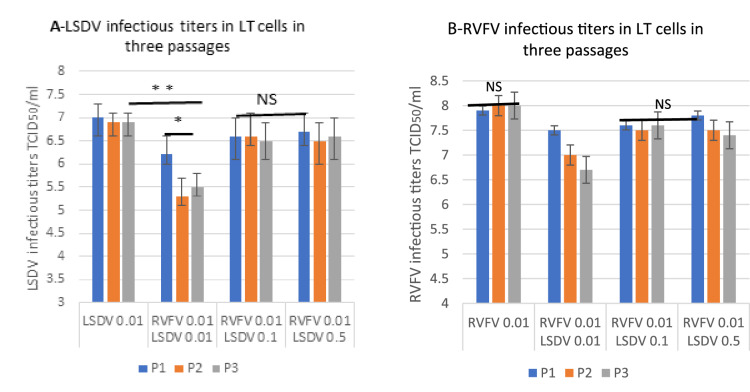
Infectivity titration (TCID_50_/ml) of LSDV (A) and RVFV (B) alone and after coinfection involving three successive passages (P1, P2 and P3) in LT cells. LSDV and RVFV alone at MOI = 0.01; and coinfection at different MOIs: RVFV/LSDV:0.01/0.01; 0.01/0.1 and 0.01/0.5. Data shown are means of at least three independent experiments (n = 3). NS: not significant. * statistically significant p < 0.05.

To determine if interference of the viruses occurred in LT cells, the cells were co-infected with SPPV/LSDV and RVFV at different MOI as shown in Figs. 3 and 4. Results showed that coinfection with similar MOI (0.01/0.01) caused a significant drop in the infectious titer of SPPV by about 1 log_10_ TCID_50_ (p = 0.0035) after three passages (Fig. 3A) when harvested after 4 days of incubation with no significant decrease in the titer of RVFV (infective titer is > 10^7^ TCID_50_/ml) (Fig. 3B). This result was confirmed by qPCR which showed the Ct to be 3 to 4 times higher between the 1st and 3nd passage especially with the poxviruses (Fig. 3A) and no significant difference in RVFV Ct in different MOI and passages (Fig. 3B). There was also a significant drop (1 log_10_ TCID_50_/ml) in the LSDV titer during passages in similar MOI as compared to the titer of the LSDV (in three passages 10^7^ ± 0.2 TCID_50_/ml) (Fig. 4A). In Fig. 4A, the titers for the RVFV 0.01 LSDV 0.01, LSDV titer appears also to be significantly lower than those for LSDV 0.01 for all three passages. However, at higher relative doses for LSDV, there does not appear to be any difference with LSDV alone. When co-infected, the titer of the RVFV decreased but not significantly (p = 0.27) (Fig. 4B). Likewise, Figs. 3 and 4 showed that when the SPPV/LSDV and RVFV were co-cultured at different MOIs (with higher starting pox viruses MOI (RVFV 0.01 and higher MOI for SPPV 0.1, 0.25, 0.5 and 1), SPPV replication was enhanced to titers of 10^6.6^ and 10^7.4^ TCID/ml during the two subsequent passages as much as when the virus was inoculated alone (Fig. 3B). These titers were confirmed by Ct values (Fig. 3B). Enhanced replication was also observed for LSDV during successive passages of a mixture of the pox viruses using higher MOIs such as 0.1 and 0.5 (Fig. 4A). The aim of these experiments was to adjust the SPPV/LSDV titer to possibly correct the interference due to RVFV faster replication. In addition, the purpose of doing the successive blind passages was to assess if pox viruses were able to replicate after three consecutive passages despite the fast replication of RVFV.

### Interference SPPV/RVF and LSDV/RVFV study in target animals: sheep and cattle

**Figure 5:**
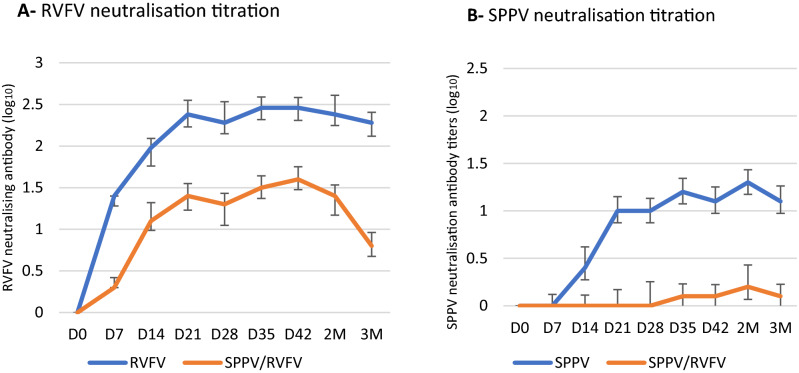
Antibody titers response of sheep injected with SPPV alone, RVFV alone, or with combined SPPV/RVFV. The antibody response was determined from from D0 to 3 months pv. (**A**) Viral neutralizing antibody titers of sheep injected with RVFV and combined SPPV/RVFV (**B**) Viral neutralizing antibody titers for SPPV Monovalent and SPPV/RVFV co-infected sheep. Data shown are means of at least three neutralization experiments. D: Days M: Months.

The neutralizing antibody response of sheep to RVFV differed in comparison to the response to a combination of SPPV/RVFV, with the response to combined viruses being lower (1.6 log_10_) than the response to RVFV alone (2.5 log_10_) (Fig. 5A). Therefore, the lower antibody response to RVFV suggested a moderate interference by the SPPV. Whereas a dramatic drop in the antibody response to SPPV response in the combined SPPV/RVFV vaccinated group (0.2 log_10_) compared to sheep vaccinated with SPPV alone (1.4 log_10_) (Fig. 5B). Thus, the antibody response to SPPV was interfered with RVFV that could be due merely to the replication of RVFV clone 13 T.

**Figure 6:**
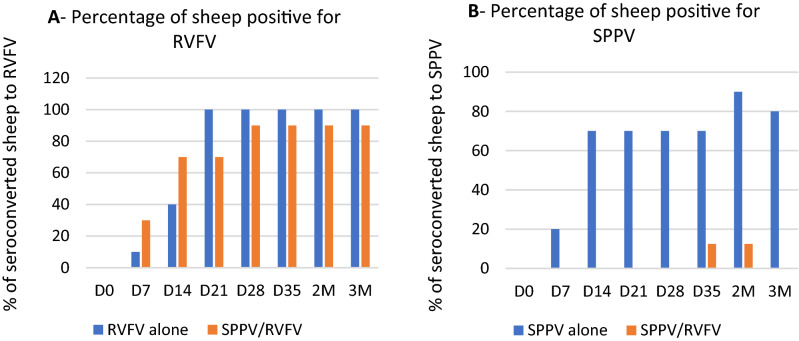
Percentage of antibody positive sheep (%) (n = 10) injected with RVFV alone, SPPV alone, or RVFV/SPPV from D0 to 3 months pv. (**A**) RVFV antibody positive sheep, (**B**) SPPV antibody positive sheep in n = 10 each of tested groups. D: Days M: Months.

Among sheep vaccinated with the combined SPPV and RVFV, 90% developed antibody to RVFV, while all sheep were seroconverted for RVFV when these animals were injected with RVFV alone (Fig. 6A). The percentage of animals that developed antibody when SPPV was injected alone was 90% at 2 months pv, whereas the development of antibody was noticed in only 10% of sheep vaccinated with the SPPV/RVFV mixture (Fig. 6B).

**Figure 7:**
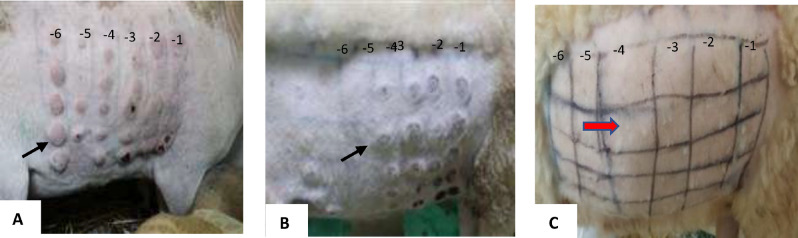
Challenged vaccinated sheep showing hypersensitivity reaction and no local inflammations on site of inoculation with10^−1^ to 10^−6^ dilutions (left to right) of virulent SPPV. (**A**) Figure of challenged unvaccinated sheep showing local inflammation on site of inoculation. (**B**) Sheep vaccinated with SPPV/RVFV and challenged with SPPV virulent strain (**C**) sheep vaccinated with SPPV alone and challenged with SPPV virulent strain. Black arrow shows inflammation or hypersensitivity reaction on inoculation site. Red arrow shows absence of the hypersensitivity reaction.

The sheep that were challenged with a virulent SPPV strain after vaccination with the combination of SPPV/RVFV were not protected (Fig. 7B); these sheep showed local inflammation at the sites of inoculation that looked similarto the unvaccinated group after challenge with virulent strain (Fig. 7A). Whereas vaccination with SPPV alone conferred full protection (Fig. 7C). Unvaccinated animals and those vaccinated with SPPV/RVFV combination developed skin lesions followed by the development of purulent papules with a peak of 41 °C of hyperthermia on D5 pi (data not shown). Thus, these observations showed that sheep vaccinated with the RVFV/SPPV combination were not protected against challenge with the virulent strain of SPPV.

**Figure 8:**
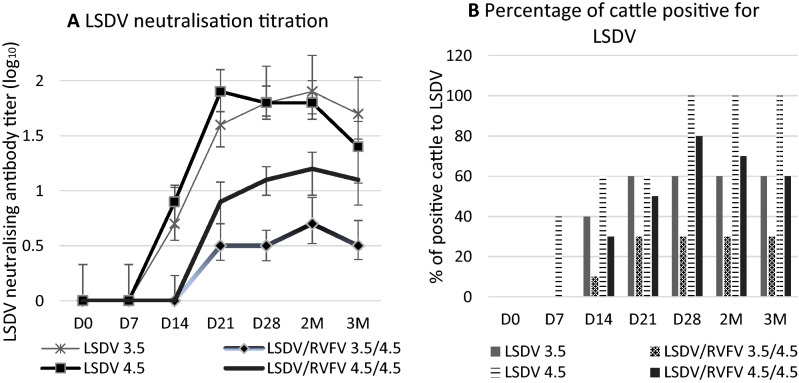
Antibody titers response in cattle vaccinated with LSDV and with combined LSDV/RVFV monitored from D0 to 3 months pv. (**A**) LSDV neutralization antibody titers in log_10_. Data shown are means of at least three neutralization experiments. (**B**) Percentage of cattle positive for LSDV injected with two different doses for LSDV (10^3.5^ and 10^4.5^ TCID_50_/dose) and RVFV (10^3.5^ and 10^4.5^ TCID_50_/dose). D: Days M: Months.

A significant drop in neutralizing antibody response against LSDV was observed in cattle vaccinated with the LSDV/RVFV combination as compared to the vaccination of cattle with LSDV alone (Fig. 8A). In cattle vaccinated with 10^3.5^ TCID_50_/dose of LSDV, the antibody response was significantly higher to monovalent LSDV (1.9 log_10_) than to the combined LSDV/RVFV (0.7 log_10_). The percentage of animals that seroconverted to LSDV was 60% vs 30 to 40% for the LSDV/RVFV combination (Fig. 8B). When we increased the LSDV dose to 10^4.5^ rather than 10^3.5^, neutralizing antibody titer improved (1.3 log_10_) but was still lower than that of the monovalent vaccine (Fig. 8A). The percentage of positive cattle vaccinated with the 10^4.5^ TCID_50_/dose of LSDV/RVFV combination was 50 to 80%, in contrast to 100% of the animals that were vaccinated with LSDV alone (Fig. 8B).

**Figure 9:**
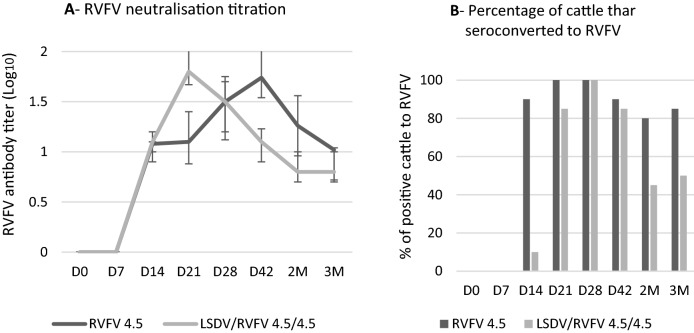
Antibody titers response in cattle vaccinated with RVFV alone, and with combined RVFV/LSDV vaccine. Cattle are monitored from D0 to 3 months pv. (**A**) RVFV neutralization antibody titers. Data shown are means of at least three neutralization experiments. (**B**) Percentage of cattle thar seroconverted to RVFV injected with a dose of 10^4.5^ TCID_50_/dose and with a dose of LSDV/RVFV 10^4.5^ TCID_50_/dose. D: Days M: Months.

Among the cattle vaccinated with the RVFV alone and with the RVFV/LSDV combination, 100% developed antibody to RVFV with antibody titers as high as 1.7 log_10_ and 1.8 log_10_, respectively (Fig. 9A and B). However, the RVFV antibody positive animals decreased at 2 and 3 months after vaccination with LSDV/RVFV. This result suggested that the LSDV interfered with the antibody response to RVFV vaccination (albeit lower than the one of RVFV over LSDV).

Interferon Gamma using Bovigam assay was used to assess cellular immunity response to LSDV. Table 1 showed that 85% of animals (17 out of 20 cattle) had a positive IFNG reaction to LSDV vs 30% (6 out of 20) in co-infected LSDV/RVFV group.

**Table 1:** Interferon Gamma Bovigam assay in cattle vaccinated with LSDV and RVFV/LSDV.

Inoculum	LSDV titer TCID_50_/dose	Positive cattle	% positive to IFNG
LSDV (n = 20)	10^5.0^	17/20	85
LSDV/RVFV (n = 20)	10^5.1^	6/20	30

## Discussion

The overall goal of this study was to evaluate the possible interference between RVFV clone 13 T and two capripoxviruses, SPPV and LSDV. RVF, SPP and LSD are economically devastating diseases of sheep and cattle with different epidemiological situations^16–18^. Vaccination is the most efficient tool to control those pathogens; moreover, multivalent vaccines present many advantages compared to monovalent products. In fact, multivalency is an important feature of an ideal vaccine, especially when a one-shot vaccination induces immunity against various infections^19^. Understanding interactions between viral pathogens is essential for the development of effective combined vaccines.

In order to evaluate combined capripoxviruses/RVFV vaccines, we investigated the coinfection with both SPPV/RVFV and LSDV/RVFV on susceptible cells and assessed the immune response of these combinations in target animals as a potential combined live-attenuated vaccine candidate. We are not aware of any available work on combined capripoxviruses/RVFV live-attenuated vaccine; however, data have been recently published on recombinant bivalent capripoxviruses/RVFV vaccine. The developed bivalent capripoxvirus/RVFV (rKS1/RVFV) expressed RVFV Gn and Gc glycoproteins^20^. This recombinant vaccine protected sheep against RVFV and SPPV challenge. Also, a bivalent LSDV-vectored RVFV for cattle was developed in which the thymidine kinase gene was used as the insertion site for the Gn and this bivalent recombinant vaccine was evaluated in cattle for its ability to provide protection against both virulent LSDV and RVFV challenge^21^. However, the very high cost is a major disadvantage of recombinant vaccines. In contrast, live-attenuated vaccines are cheap and affords satisfactory protection when herd immunity is sustained by carrying out annual vaccinations^18^. This study is the first work that illustrates interference between RVFV and capripoxviruses attenuated strains in cell culture and in target animal species during the testing of these combined viruses.

Few in-vitro and in-vivo studies have been conducted in order to determine mechanisms of possible interactions when two or more viral components have resulted in the development of live-attenuated veterinary vaccines. Some viruses could suppress other viruses replication; this phenomenon is called viral interference^22,23^. Interference could be either homologous or heterologous. Heterologous viral interference describes a negative interaction between viruses from different families^24^. On the other hand, successful combination between two viral antigens was also demonstrated. No interference was noticed during coinfection by PPRV and SPPV in LT cells^25^. In fact, this combination between PPRV and SPPV led to the production of a successful vaccine in our lab^8^.

In vitro coinfection interference of capripoxviruses and RVFV was evaluated by the appearance of CPE. Capripoxviruses induced vacuoles in cells and intracytoplasmic eosinophilic inclusion bodies (typical capripoxviruses CPE profile)^26,27^. These bodies spread slowly on the cell monolayer, whereas, RVFV CPE was characterized by dead floating cells that were visibly detaching from the monolayer in LT cells much faster than pox viruses. RVFV CPE appeared as necrotic foci especially when the MOI of capripoxviruses was lower or similar to RVFV MOI (0.01/0.01 for instance). Vero cells were not suitable to study the coinfection of both viruses, especially for SPPV. Even at high MOI (MOI = 1), SPPV did not replicate efficiently (10^5.2^ TCID_50_/ml) while RVFV clone 13 T successfully replicated regardless of the MOI used to infect the cells. LT cells were selected for coinfection of SPPV and RVFV because these cells were more susceptible to capripoxviruses^25,26^. Also, the simultaneous replication of both viruses was better in LT cells than in Vero cells but there was a slightly better replication in favor of pox viruses when the MOI was high, whereas RVFV replicated better when SPPV were inoculated at low MOI (0.001). According to Kumar et al., the rate of virus’s replication and CPE formation were considered as factors influencing outcome of coinfections^28^. Indeed, cytolytic viruses could rapidly deplete cellular resources and induce cell death as we observed for RVFV clone 13 T and SPPV coinfection of cells. Therefore, if coinfecting viruses vary significantly in the length of their replication cycle, the one with the shorter replication cycle will persist because the second virus with the longer replication cycle will be prematurely terminated.

In order to determine if capripoxviruses and RVFV replicated efficiently and sustainably together during successive passages in LT cells at similar MOI (0.01/0.01), we determined the infectivity titers along with copies of viral RNA using quantitative PCR. Despite satisfactory infectivity titers (> 10^6.5^ TCID_50_/ml), obtained when both viruses were co-infected with same low MOI (0.01/0.01), we noticed a significant drop in the infectivity titers after 3 blind passages of the poxviruses compared to titers obtained when these viruses infected cells alone. The decrease in titer during passages was observed for both viruses using similar MOI (0.01/0.01). Results were also confirmed by quantitative PCR at different MOI and passages. As the passages increased, infectivity titers of SPPV and LSDV tended to decrease. This could be explained by the pattern of RVFV replication that starts earlier after infection while capripoxviruses replication begins 2 to 5 h pi^4,29,30^. When the MOI (0.1, 0.25, and 0.5) of SPPV or LSDV was increased as compared to and MOI of 0.01 for RVFV MOI for infecting LT cells, capripoxvirus’ replication improved. Indeed, the purpose of this experiment was to be able to correct the respective titers when both viruses are combined.

Virus accumulation in cell culture depends on the infection dose per cell that mainly affects the length of the virus replication. The MOI dose of 0.001–0.1 is usually used to infect monolayer cultures in order to accumulate the virus. When using similar MOI for both viruses, RVFV seems to interfere negatively with capripox virus’ replication. Poxvirus replication occurs in the cytoplasm within distinct juxtanuclear sites named factories^4^. A poxvirus factory can be formed from a single virion, and the number of factories is proportional to the MOI^4,23,30^. In our study, we adsorbed capripoxvirus for 45 min before inoculating RVFV to promote poxvirus replication. The virus accumulation is the sum result of multi cycle reproduction. As both capripox and RVF viruses replicate in the cytoplasm, the existence of mutual inhibition of replication suggested that the two viruses could not co-replicate in one biosystem. Indeed, viral interference could be directly influenced by both virus doses (MOI) and the time interval between co-infecting viruses^28^. Singh et al., reported that when wild-type and mutant strains of Semliki forest virus (SFV) were added together at an MOI of 5, all the cells became infected, but if wild-type SFV was added 15 min after, fewer than 30% of cells were infected with second virus^31^. In another study, instead of coinfection, infection with foot-and-mouth disease virus at 12 h after PPRV induced viral interference^32^. In vaccinia virus superinfection, the secondary virus could not replicate at all if it was applied 4 h later^33^.

When we used combined SPPV/RVFV attenuated viruses to vaccinate target animals, SPPV/RVFV combination failed to protect sheep against SPPV infection and LSDV/RVFV combination did not succeed to produce similar immunological response as the monovalent LSDV vaccine in cattle. The antibody response to SPPV and LSDV when combined with RVFV was significantly lower in comparison with the response after monovalent SPPV or LSDV injection of the animals. Indeed, sheep did not resist the experimental infection by virulent strain and cattle did not react positive to IFNG, test of cellular immunity. Immunity response to LSDV is known to be predominantly cell mediated^34^. The interference also affected the RVFV antibody response but not as much as the effect on the capripoxviruses’ response. In fact, RVFV neutralizing antibody titers in sheep was 1.7 log_10_ following vaccination with the combined SPPV/RVFV vaccine versus 2.5 log_10_ after vaccination with RVFV alone. Despite this slight drop, neutralizing antibody titers remain > 1.5 log_10_ which is equivalent to protection^35^. This interference could be explained by the short replication cycle of RVFV that takes advantage of the longer SPPV/LSDV replication cycle. Also, it has been reported that viral interference could be mediated by several factors, such as interferons (IFNs), defective interfering (DI) particles, cellular factors, and others^23^.

In our study, clear interference was detected between RVFV clone 13 T and both SPPV and LSDV attenuated viruses. The RVFV attenuated virus interfered with capripoxvirus replication upon their coinfection of cell culture and in vivo when tested in target animals: sheep and cattle. Interference among attenuated live vaccine strains was reported to occur between enteroviruses and poliovirus vaccine that resulted in vaccine failure^36^. The interference was due to the production of antibody which constrains the growth of the secondary virus upon vaccination^28,37^. Viral interference has been shown for other vaccines and viruses, such as Newcastle disease virus (NDV) in chickens, infectious bronchitis virus and NDV vaccine strains^38^ and experienced with diverse groups of viruses such as dengue virus^39^. Consequently, understanding viral interference is of an extreme importance for the formulation of any successful vaccine combination^40^.

This report is an example of heterologous interference between RVFV, an RNA virus and both SPPV and LSDV, two DNA viruses. The capacity of an RNA virus to interfere with a DNA virus raises the intriguing question of whether competitive inhibition can occur between nucleic acids with presumed dissimilar metabolic pathways^41^. There is insufficient evidence to implicate genetic or metabolic factors as explanations for competitive antagonism between nucleic acid moieties of two viruses within the same cell^41^. However, the nucleic acid of the interfering virus may well be essential for initiating the cellular response that leads to interference. It was reported that RNA viruses produce defective interfering (DI) particles at high MOI serial passages in cell culture^42,43^. DI are spontaneously produced defective genomes virus mutants^44^. Moreover, these deficient viral particles replicate faster than the standard virus^45^. Abnormal defective RNAs have also been described for RVFV^46^.

In conclusion, this study opens new insights toward studying the reason(s) behind immunity inhibition of capripoxviruses attenuated strains in the presence of RVFV lacking NS gene (clone 13). It was clearly shown that injection of SPPV and LSDV in the presence of RVFV clone 13 T was counteracted. Indeed, in this study we showed that sheep were not protected when co-injected with SPPV/RVFV and neither LSDV immunity (humoral nor cellular) was induced in cattle when co-injected with LSDV/RVFV. Co-inoculation with RVFV and SPPV/LSDV on susceptible cell culture revealed interference between RVFV and SPPV and/or LSDV. Further investigations should be done in order to elucidate and comprehend the mechanism behind this “interference” in sheep, cattle and in goats using goatpox virus GTPV (Gorgan strain), where there is also a clear interference between RVFV and GTPV when challenged with Vietnamien virulent strain (data not shown). Two strategies could be suggested a) to increase the SPPV titer and decrease RVFV infectivity titer/dose for combined SPPV/RVFV infection. Then, to evaluate the immune response of sheep co-injected with 10^4.5^ TCID_50_/dose for SPPV and 10^3.0^ TCID_50_/dose for RVFV. Whereas, for cattle, to increase the LSDV titer (10^5.5^ TCID_50_/dose) and decrease RVFV to (10^3.5^ TCID_50_/dose). b) Since capripoxviruses use lymphatic route of infection and RVFV spread through blood, two separate injections of the SPPV alone in one side and the monovalent RVFV in another site in sheep should be considered in order to avoid this interference.
